# Clinical factors influencing long-term survival in a real-life cohort of early stage non-small-cell lung cancer patients in Spain

**DOI:** 10.3389/fonc.2023.1074337

**Published:** 2023-02-23

**Authors:** Maria Torrente, Pedro A. Sousa, Gracinda R. Guerreiro, Fabio Franco, Roberto Hernández, Consuelo Parejo, Alexandre Sousa, José Luis Campo-Cañaveral, João Pimentão, Mariano Provencio

**Affiliations:** ^1^ Department of Medical Oncology, Puerta de Hierro-Majadahonda University Hospital, Madrid, Spain; ^2^ Faculty of Health Sciences, Francisco de Vitoria University, Madrid, Spain; ^3^ Department of Electrical Engineering, NOVA School of Science and Technology, Universidade Nova de Lisboa, Lisbon, Portugal; ^4^ Department of Mathematics and CMA, NOVA School of Science and Technology, Universidade Nova de Lisboa, Lisbon, Portugal; ^5^ Department of Thoracic Surgery, Puerta de Hierro-Majadahonda University Hospital, Madrid, Spain

**Keywords:** non-small cell lung cancer, risk stratification, prognostic model, early stage, long-term survival

## Abstract

**Background:**

Current prognosis in oncology is reduced to the tumour stage and performance status, leaving out many other factors that may impact the patient´s management. Prognostic stratification of early stage non-small-cell lung cancer (NSCLC) patients with poor prognosis after surgery is of considerable clinical relevance. The objective of this study was to identify clinical factors associated with long-term overall survival in a real-life cohort of patients with stage I-II NSCLC and develop a prognostic model that identifies features associated with poor prognosis and stratifies patients by risk.

**Methods:**

This is a cohort study including 505 patients, diagnosed with stage I-II NSCLC, who underwent curative surgical procedures at a tertiary hospital in Madrid, Spain.

**Results:**

Median OS (in months) was 63.7 (95% CI, 58.7-68.7) for the whole cohort, 62.4 in patients submitted to surgery and 65 in patients submitted to surgery and adjuvant treatment. The univariate analysis estimated that a female diagnosed with NSCLC has a 0.967 (95% CI 0.936 - 0.999) probability of survival one year after diagnosis and a 0.784 (95% CI 0.712 - 0.863) five years after diagnosis. For males, these probabilities drop to 0.904 (95% CI 0.875 - 0.934) and 0.613 (95% CI 0.566 - 0.665), respectively. Multivariable analysis shows that sex, age at diagnosis, type of treatment, ECOG-PS, and stage are statistically significant variables (p<0.10). According to the Cox regression model, age over 50, ECOG-PS 1 or 2, and stage ll are risk factors for survival (HR>1) while adjuvant chemotherapy is a good prognostic variable (HR<1). The prognostic model identified a high-risk profile defined by males over 71 years old, former smokers, treated with surgery, ECOG-PS 2.

**Conclusions:**

The results of the present study found that, overall, adjuvant chemotherapy was associated with the best long-term OS in patients with resected NSCLC. Age, stage and ECOG-PS were also significant factors to take into account when making decisions regarding adjuvant therapy.

## Introduction

Lung cancer is the worldwide leading cause of cancer-related mortality, with non-small cell lung cancer (NSCLC) accounting for approximately 85% of all lung cancer patients. Owing to the tendency for late diagnosis and tumour recurrence (1, 2), the 5-year overall survival (OS) rate for NSCLC remains low at about 23% and significantly varies by stage, with 5-year OS rates being as high as 73% for patients with stage IA and as low as 2% for those with stage IV disease (3, 4). As a result, there has been considerable effort to identify patients with early-stage NSCLC who may benefit from additional treatment after surgery. Adjuvant radiotherapy is no longer recommended after surgery because it has been shown to have a deleterious effect on long-term survival, at least for stage I and II disease (5). Current European Society for Medical Oncology (ESMO) guidelines for early-stage NSCLC clearly indicate surgery for stages I and II, with adjuvant chemotherapy recommended for stage II and considered for stage IB. Radiotherapy is recommended as a nonsurgical option for stage I (6).

Current prognosis in oncology is reduced to the tumour stage and performance status of the patients, leaving out many other factors that may impact the patient´s management. Even if a few, more advanced stratification models for cancer patients have been proposed, these are usually focused on very specific typologies and require analyses not commonly available in the clinical practice (7) or have not been validated in multiple international cohorts (8–10). Smarter stratification models that leverage data about disease interactions, disease severity, and treatment pathways based on electronic health records (EHRs) can provide crucial information for making better clinical decisions about patients with cancer. Therefore, prognostic stratification of patients with poor prognosis after surgery, in order to assist physicians to make decisions on therapeutic strategies is of considerable clinical relevance.

We here report the results of a study aimed to identify clinical factors associated with long-term overall survival in a real-life cohort of patients with stage I-II NSCLC treated at a tertiary hospital in Madrid, Spain, and develop a prognostic model that identifies poor prognosis factors and stratifies patients by risk.

## Methods

### Data source

This cohort study used data obtained from a hospital-based lung cancer registry managed by the Department of Medical Oncology at Puerta de Hierro-Majadahonda University Hospital (HUPHM). It is a structured database registered in the RedCap platform, that collects de-identified clinical data from lung cancer patients at HUPHM. The study was approved by the Ethics Committee at HUPHM (No. PI 148/15) and was carried out in accordance with the Helsinki Declaration.

### Study population

This is a hospital-based retrospective study that updates prospective follow-up data in the population of NSCLC patients diagnosed and treated at HUPHM from 2008, regardless of their treatment, sex, or age. The last follow-up or vital status information was updated in December 2021. All patients included underwent curative surgery as primary treatment and had pathological confirmation on surgical sample of NSCLC in early stages (I–II). The exclusion criteria were: performed neoadjuvant therapy; unavailable clinicopathological, vital status, or follow-up data; and age < 18 years old.

**Figure 1:**
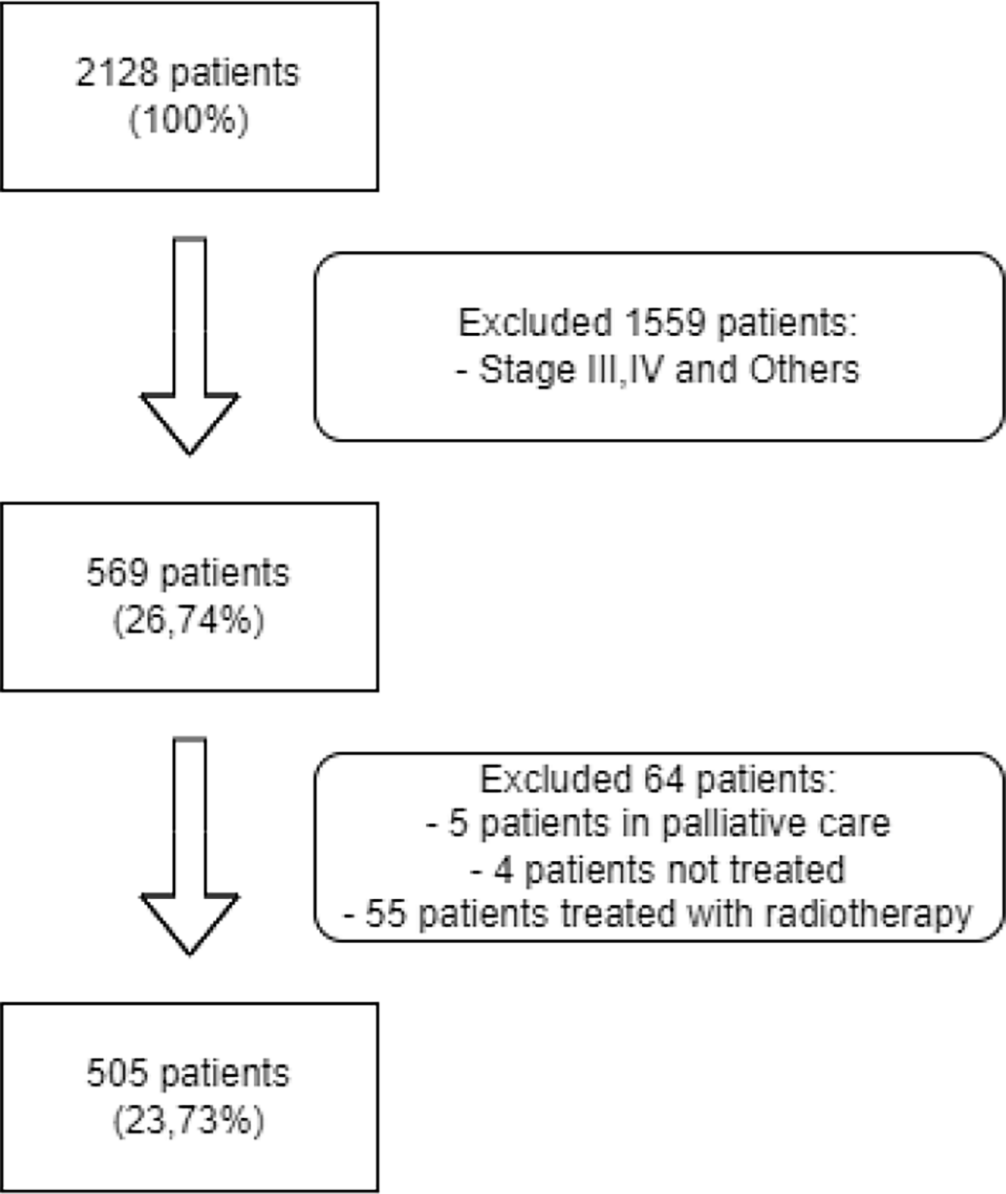
Flow Diagram for patient selection.

Clinical data from 2128 patients was extracted from the EHR and structured in a dataset (**Figure 1**). Of those, 1559 were excluded due to diagnosis of metastatic disease (stage III and IV). Additionally, 55 patients who received radiotherapy as primary treatment were excluded from the study.

### Study variables

Patient, tumour, and treatment characteristics were collected from the EHR and structured in RedCap platform: demographic parameters, performance status (ECOG-PS, Eastern Cooperative Oncology Group-Performance status), tobacco habit, comorbidities, family history of cancer, histologic type, disease stage (patients were staged according to seventh edition of TNM classification by American Joint Committee on Cancer (AJCC7) and reclassification using AJCC eighth edition (AJCC8) was also performed), treatments (surgery, adjuvant chemotherapy), and relapse of the disease. Smoking status was defined as never smoker, former smoker, and current smoker. Smokers who claimed to have quit in the 8 weeks prior to diagnosis were classified as current smokers.

### Statistical analysis

Statistical analysis was performed using R Software, version 4.0.5. Quantitative data were expressed as mean, median and standard deviation (SD). The qualitative variables were expressed in the form of frequencies and percentages. Univariate and multivariate analyses were conducted to evaluate the primary patients’ characteristics leading to better OS. Univariate survival analysis was performed using Kaplan-Meier curves and survival functions were compared using a log-rank test to check for differences. Statistical significance for the log-rank test was set at p<0.05. To investigate the contribution of each characteristic in the survival time, Cox Multivariate regression model was adjusted using a backward stepwise procedure. Significance level was set at p<0.10. The assumption of the proportionality of hazards was evaluated with Schoenfeld residuals. Survival Time for Stages I and II patients has a long right tail distribution, with outliers representing long survivors. Using univariate Kaplan Meier analysis, the probabilities of survival after 12 and 60 months were estimated (Estimate, SE, lower and upper 95% confidence interval). Disease free survival (DFS) was calculated from the date of surgery until the date of death or relapse. Using the Kaplan-Meier estimator, the OS curve and the DFS for both Stage I and II were estimated.

## Results

### Patient characteristics

A total of 505 patients with stage I and II NSCLC were included. The baseline characteristics of the patients are detailed in **Table 1**. Overall, there was a significantly greater number of men (76%) compared to women (24%). The median age at diagnosis was 60.6 years; 64% of the patients were aged between 51 and 70 years; 5.1% were under 50 and 31% over 71. Regarding smoking habits, 55.6% of the diagnosed patients were former smokers and 31.7% current smokers, with only 10.7% of never smokers. Most of the patients were diagnosed in stage I (62.8%) compared to stage II (37.2%); 96% of patients had an ECOG-PS of 0 or 1, 80% received surgery as primary treatment, while 20% received surgery plus adjuvant treatment, and 89% had comorbidities. Finally, in this patient cohort, 32.3% of patients relapsed during the follow-up period and received subsequent therapy.

**Table 1:** Characteristics of the patients.

	All Cohort						
Characteristics	Total	%	Survival Mean			Deceased	
			Months	Median	SD		
Overall	505	100%	63,7	61,9	39,1	219	43%
Gender							
Female	121	24,0%	66,7	69,8	38,9	36	29,8%
Male	384	76,0%	60,3	59,5	38,8	183	47,7%
Age at Diagnosis [years}							
20-50	26	5,1%	73,3	88,8	65,9	7	26,9%
51-7O	323	64,0%	65,7	62.6	37,2	139	43,0%
71+	156	30,9%	59,9	56,1	35,0	73	46,8%
Smoking Habits							
Non Smoker	54	10,7%	70,8	78,1	38,7	14	25,9%
Former Smoker	281	55,6%	56,2	58,1	42,6	144	51,2%
Current Smoker	160	31,7%	65,6	63.0	31,2	58	36,3%
Unknown	10	2,0%	-	-	-	3	30,0%
Stage							
I	317	62,8%	67,7	64,6	33,1	120	37,9%
II	188	37,2%	45,1	57,5	47,2	99	52,7%
Comorbidities							
No	56	11,1%	63,9	67,8	45,3	22	39,3%
Yes	449	88.9%	63,2	61,2	38,2	197	43,9%
Patient with Previous Cancer							
No	343	67,9%	64,0	63,4	39,5	145	42,3%
Yes	149	29,5%	62,5	60,1	33,2	67	45,0%
Unknown	13	2,6%	-	-	-	7	53,3%
Treatment							
Surgery	402	79,6%	62,4	60,4	38,8	186	46,3%
Surgery + Adjuvant CHT	103	20,4%	65,0	67,9	39,5	33	32,0%
Performance status							
0	355	70,3%	66,8	63,2	32,7	117	33,0%
1	131	25,9%	44,4	61,5	53,0	90	68,7%
2	8	1,6%	16,0	28,4	25,6	7	87,5%
Unknown	11	2,2%	-	-	-	5	45,5%
Relapse							
No disease	226	44,75%	69,1	67,7	36,2	58	25,7%
Relapse/Progression	163	32,28%	42,7	54,7	43,4	114	69,9%
Unknown	116	22,97%	-	-	-	47	40,5%

CHT, chemotherapy; SD, standard deviation.

### Survival analysis

#### Overall survival of the whole population

**Figure 2:**
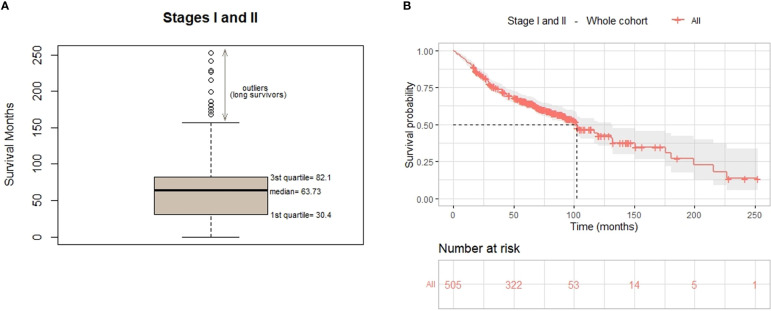
Overall survival of the whole cohort. **(A)** Box and whisker plot shows median AND quartiles survival (in months) and outliers. **(B)** OS curve for the 505 early stage patients estimated using the Kaplan-Meier estimator.

Median survival in our cohort was 63.7 months (95% CI, 56.7-64.4) (**Figure 2A**); 25% of the patients have a survival time less than 30.4 months, and the 3rd quartile indicates that the survival time of 25% of the patients is higher than 82 months. We can also observe that 14 patients are outliers, with the highest survival. In **Figure 2B**, the curve median estimator shows the influence of these patients, alive after more than 150 months since diagnosis. As illustrated by the CI amplitude, the statistical relevance above 120 months decreases due to the reduced number of patients. The observed survival time of long survivors has an impact on the Kaplan-Meier estimate for median time (**Figure 2B**) which differs significantly from the observed median of the dataset (**Figure 2A**).

Overall, 8% of the patients died within the first year since diagnosis, and 86% had a long-term OS (alive more than 2 years since diagnosis). Of note, 6% survived more than 10 years since diagnosis (**Supplementary Material**).

### Disease-free survival

**Figure 3:**
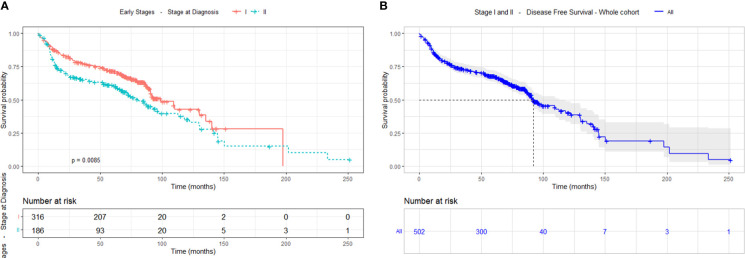
Disease-free survival, **(A)** by cancer stage (I-II) and **(B)** of the whole cohort.

There was statistical difference (p=0.0085) in DFS between stage I and II, being the median DFS for stage I 98.87 months (8.23 years) and 81.07 months (6.76 years) for stage II. The median DFS for the whole cohort was 92.23 months (7.68 years). The 5-year DFS for the stage I cohort was 63% and for stage II group was 48% (**Figure 3**).

### Results of the univariate analysis

**Figure 4:**
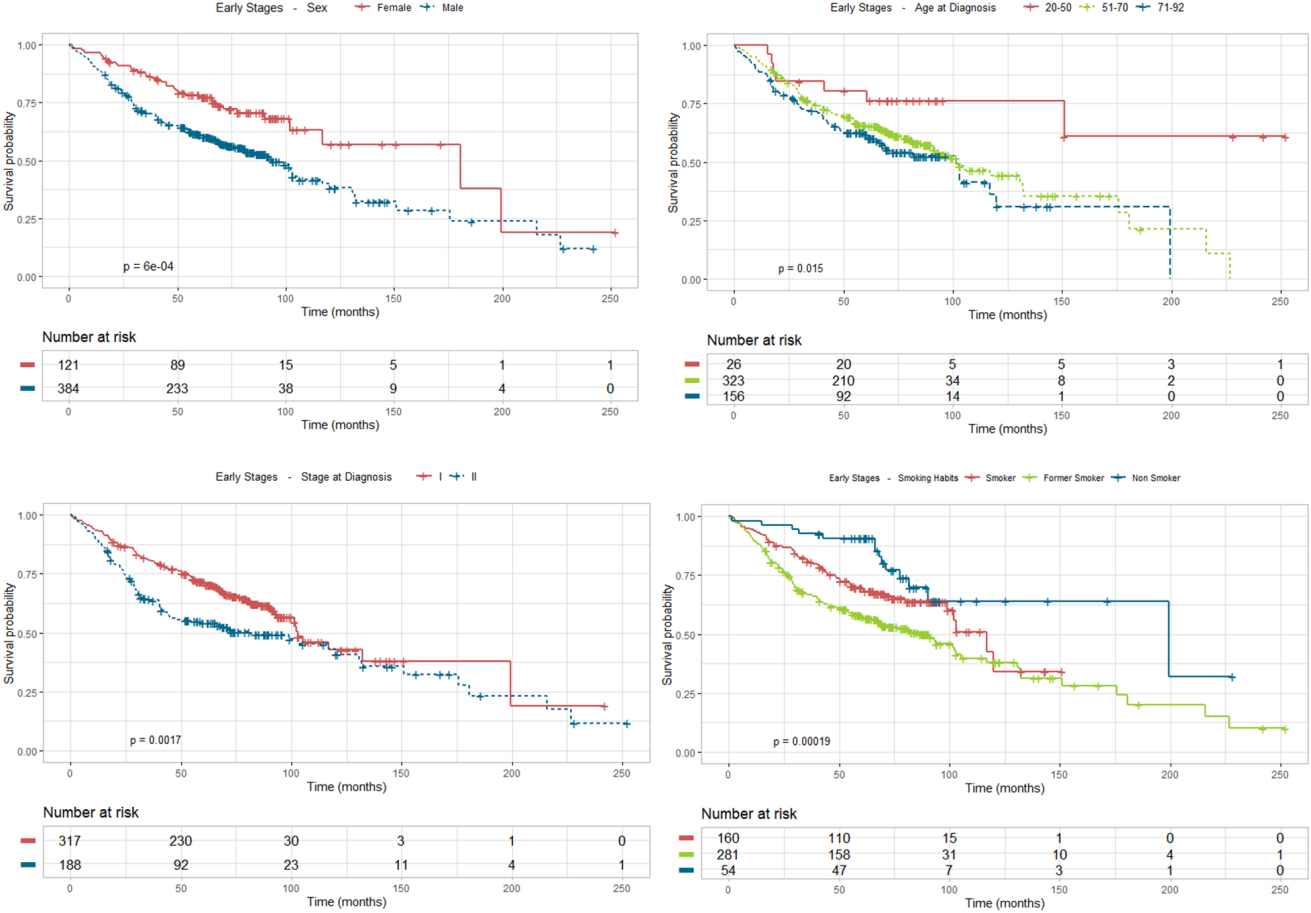
Survival analysis using Kaplan Meier estimates in stages I and II according to sex, age at diagnosis, stage and smoking habit.

The univariate analysis was performed based on survival to relate the different socio-demographic variables, as well as those related to the tumour and the type of treatment received. The analysis revealed statistically significant differences (**Figure 4**) according to sex (p<0.001), with a greater survival in women; age at diagnosis (p=0.015), with greater survival in the group of 20 to 50 years old; smoking habits (p<0.001) where survival drops dramatically in former and current smokers compared to never smokers; and stage (p=0.002), with greater survival in stage I compared to stage ll.

**Figure 5:**
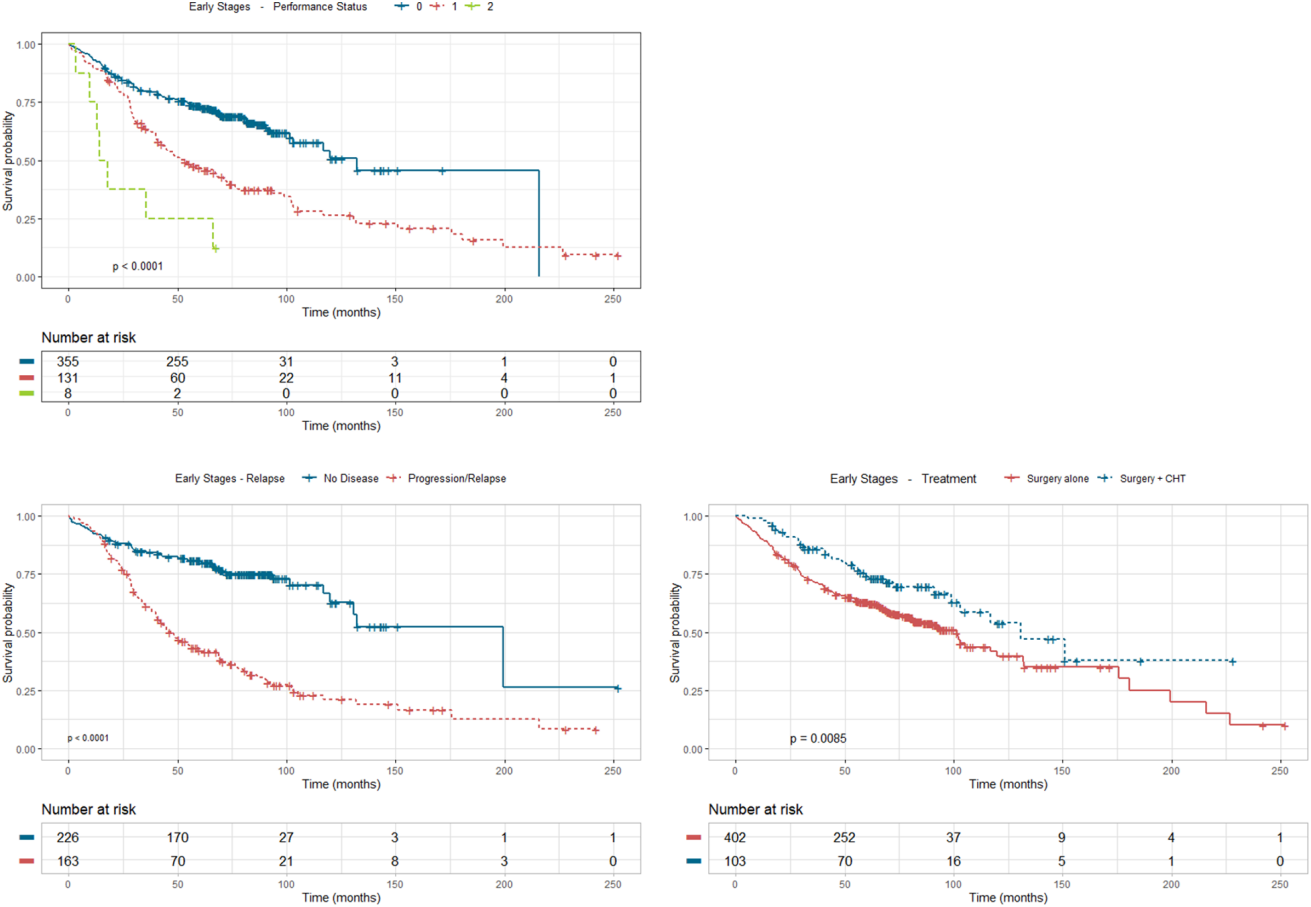
Survival analysis using Kaplan Meier estimates in stages I and II according to treatment, performance status and relapse.

As for treatment received (**Figure 5**), survival is strongly improved by surgery with adjuvant chemotherapy compared to surgery (p=0.0085). Apart from the two pivotal prognostic factors, stage and treatment, ECOG-PS also stands as a statistically significant factor that impacts prognosis (p<0.001), especially ECOG-PS 1 and 2 (although the representation of this last group is very scarce), compared to ECOG-PS 0, along with relapse of the disease (p<0.0001), which lowers dramatically patient survival, compared to disease-free status.

Using Kaplan-Meier survival curves we are able to estimate the probability of a patient surviving a given time period, in a univariate approach. The univariate analysis has different sample dimensions for each characteristic because all the patients with *unknown* value are discarded. Estimates for the probability of survival at 1 and 5 years, for each of the considered covariates, as well as the corresponding 95% CI, are shown in **Table 2**. From a univariate point of view, we estimate that a female diagnosed with NSCLC has a 0.967 (95% CI 0.936 - 0.999) probability of survival one year after diagnosis and a 0.784 (95% CI 0.713 - 0.863) five years after diagnosis. For males, these probabilities drop to 0.903 (95% CI 0.875 - 0.933) and 0.613 (95% CI 0.566 - 0.665), respectively.

**Table 2:** Univariate analysis of survival probability using Kaplan Meier estimates after 12 months and after 60 months.

Characteristics	Total	%	log-rank test		Survival Probability after 12 months				Survival Probability after 60 months		
			p-value	Estimate	St. Error	lower 95%	upper 95%	Estimate	St. Error	lower 95%	upper 95%
Overall	505	100%		0,919	0,0122	0,895	0,943	0,645	0,0216	0,613	0,697
Gender	506		<0.001								
Female	121	23,96%		0,9669	0,0163	0,9356	0,9993	0,7840	0,0384	0,7122	0,8629
Male	384	76,04%		0,9036	0,0151	0,8746	0,93336	0,6132	0,0253	0,5656	0,6648
Age at Diagnosis (years)	505		0,015								
20-50	26	5,15%		1	0	1	1	0,8060	0,0780	0,6670	0,9740
51-70	323	63,96%		0,9288	0,0143	0,9012	0,9573	0,6585	0,0269	0,6078	0,7135
71+	156	30,89%		0,8846	0,02560	0,8359	0,9362	0,6184	0,0396	0,5455	0,7011
Smoking Habits	495		<0.001								
Non Smoker	54	10,91%		0,9815	0,0183	0,9462	1	0,9066	0,0398	0,8319	0,9880
Former Smoker	281	56,77%		0,8932	0,0184	0,8579	0,9301	0,5770	0,0302	0,5207	0,6393
Current Smoker	160	32,32%		0,9375	0,0191	0,9007	0,9758	0,697	0,0371	0,628	0,7735
Stage	505		0,0017								
I	317	62,77%		0,9340	0,0140	0,9070	0,9620	0,7195	0,0256	0,6711	0,7714
II	188	37,23%		0,8936	0,0225	0,8506	0,9388	0,5409	0,0375	0,4722	0,6195
Comorbidities	505		0,32								
No	56	11,09%		0,9643	0,0248	0,9169	1	0,6540	0,0644	0,5391	0,7933
Yes	449	88,91%		0,9131	0,0133	0,8875	0,9396	0,6539	0,0229	0,6106	0,7003
Patient with Previous Cancer	547		0,47								
No	375	65,56%		0,9643	0,0248	0,9169	1	0,6540	0,0644	0,5391	0,7933
Yes	172	31,44%		0,9131	0,0133	0,8875	0,9396	0,6539	0,0229	0,6105	0,7003
Treatment	505		0,008								
Surgery	402	79,60%		0,9005	0,0149	0,8717	0,9302	0,631	0,0243	0,5850	0,6805
Surgery +Adjuvant CHT	103	20,40%		0,9900	9,0097	0,9720	1,0000	0,7488	0,0455	0,6597	0,8387
Perform ante status	494		<0.001								
0	355	71,86%		0,9296	0,0136	0,9033	0,9566	0,7344	0,0238	0,6891	0,7826
1	131	26,52%		0,9008	0,0261	0,8510	0,9534	0,4680	0,0450	0,3380	0,5650
2	8	1,62%		0,7500	0,1530	0,5030	1,0000	0,250O	0,1531	0,0753	0,8302
Relapse	389		<0.001								
No disease	226	58,10%		0,9248	0,0175	0,891	0,9598	0,8093	0,0265	0,7589	0,8631
Relapse/Progression	163	41,90%		0,9325	0,0I96	0,8948	0,9718	0,4238	0,0401	0,352	0,5101

ECOG-PS, Eastern Cooperative Oncology Group-Performance status; CHT, chemotherapy.

According to the p-value of the log-rank test, we can conclude that only comorbidities (p=0.1) and previous cancer (p=0.47) are not significant covariates, while all the other characteristics (p<0.05), reveal to be significant on survival, on a univariate approach. Of important note, survival probability estimates after 12 months do not vary much within each significant variable, while these estimates significantly differ after 60 months, such as relapse (0.423; 95% CI 0.352 - 0.510) vs no disease (0.809; 95% CI 0.759 - 0.863).

### Results of the multivariable analysis

Covariate inclusion of prognostic indicators was performed using a combination of two-sided Wald test and Likelihood ratio test (p<0.05) in addition to Akaike Information Criterion (AIC) and concordance (c-index) of the performed model. Multivariate Cox proportional hazards regression analysis showed that Sex, Age, Treatment, Stage and ECOG-PS were independent significant variables (p<0.10) associated with decreased OS, while sex, stage, comorbidities, and smoking habit were not (p>0.10). Nevertheless, despite not being significant probably due to the lack of data, being a non-smoker reduces 34% the risk of dying (HR=0.66; 95% CI 0.35-1.24). Model´s concordance (c-index) was 0.693.

The final Cox model indicated that, among the variables with statistical significance (**Table 3**), the one that revealed a protective or risk-lowering effect was surgery with adjuvant chemotherapy (HR=0.46; 95% CI 0.30-0.69), implying that, on average, this treatment reduces by 54% the risk of death when compared to patients who were treated with surgery alone. Of note, statistically significant variables that increase risk were age, especially in patients above 71 years old (HR=3.25; 95% CI 1.25-8.46), ECOG-PS 1 (HR=2.07; 95% CI 1.54-2.78) and 2 (HR=5.41; 95% CI 2.47-11.83), and stage ll (HR=1.46; 95% CI 1.08-1.98).

**Table 3:** Multivariate analysis - Cox regression model.

Characteristics	Coefficient		Standard Error	Lower L95%	Upper U95%	p value
Gender						
Female	Reference Category					
Male	0,3667	1,44	0,2019	0,97	2,14	0,0693
Age at Diagnosis						
20-50	Reference Category					
51-70	0,9942	2,70	0_r_4743	1,07	6,85	0,0361
71+	1,1795	3,25	0,4876	1,25	8,46	0,0156
Smoking Habits						
Current Smoker	Reference Category					
Former Smoker	0,2480	1,28	0,1636	0,93	1,77	0,1294
Non Smoker	-0,4134	0,66	0,3208	0,35	1,24	0,1975
Stage						
I	Reference Category					
II	0_r_3816	1,46	0,1545	1,08	1,98	0,0135
Treatment						
Surgery	Reference Category					
Surgery + Adjuvant CHT	-0_r_7782	0,46	0,2092	0,30	0,69	0,0002
Performance Status						
0	Reference Category					
1	0,7279	2,07	0_r_1505	1,54	2,73	<0.001
*2*	1,6883	5,41	0,3992	2,47	11,83	<0.001

ECOG-PS, Eastern Cooperative Oncology Group-Performance status; AJCC7, American Joint Committee on Cancer, seventh edition; CHT, chemotherapy.

Accordingly, we identified and integrated significant variables in the patient cohort to build a prognostic model that explains the probability of survival (**Table 4**). For prognosis of patients with early-stage NSCLC, the Cox survival model included six discriminative features. The prognostic model, see **Table 3**, identified a high-risk profile defined by males over 70 years old, former smokers, stage II, ECOG-PS 2, treated with surgery alone. These features correspond to the highest positive estimates for each of the covariates. The identified features for the low-risk profile (corresponding to the lowest negative coefficients) were being a female between 20 and 50 years old, non-smoker, treated with surgery and adjuvant chemotherapy, with an ECOG-PS 0.

**Table 4:** Prognostic model of survival including significant variables from the multivariate analysis.

Cox Survival Model		
Variables	Higher Risk Profile	Lower Risk Profile
Gender	Male	Female
Age at Diagnosis	71+	20-50
Smoking Habits	Former Smoker	Non Smoker
Stage	II	1
Treatment	Surgery	Surgery + Adjuvant CHT
Performance Status	2	0

CHT, chemotherapy.

**Figure 6:**
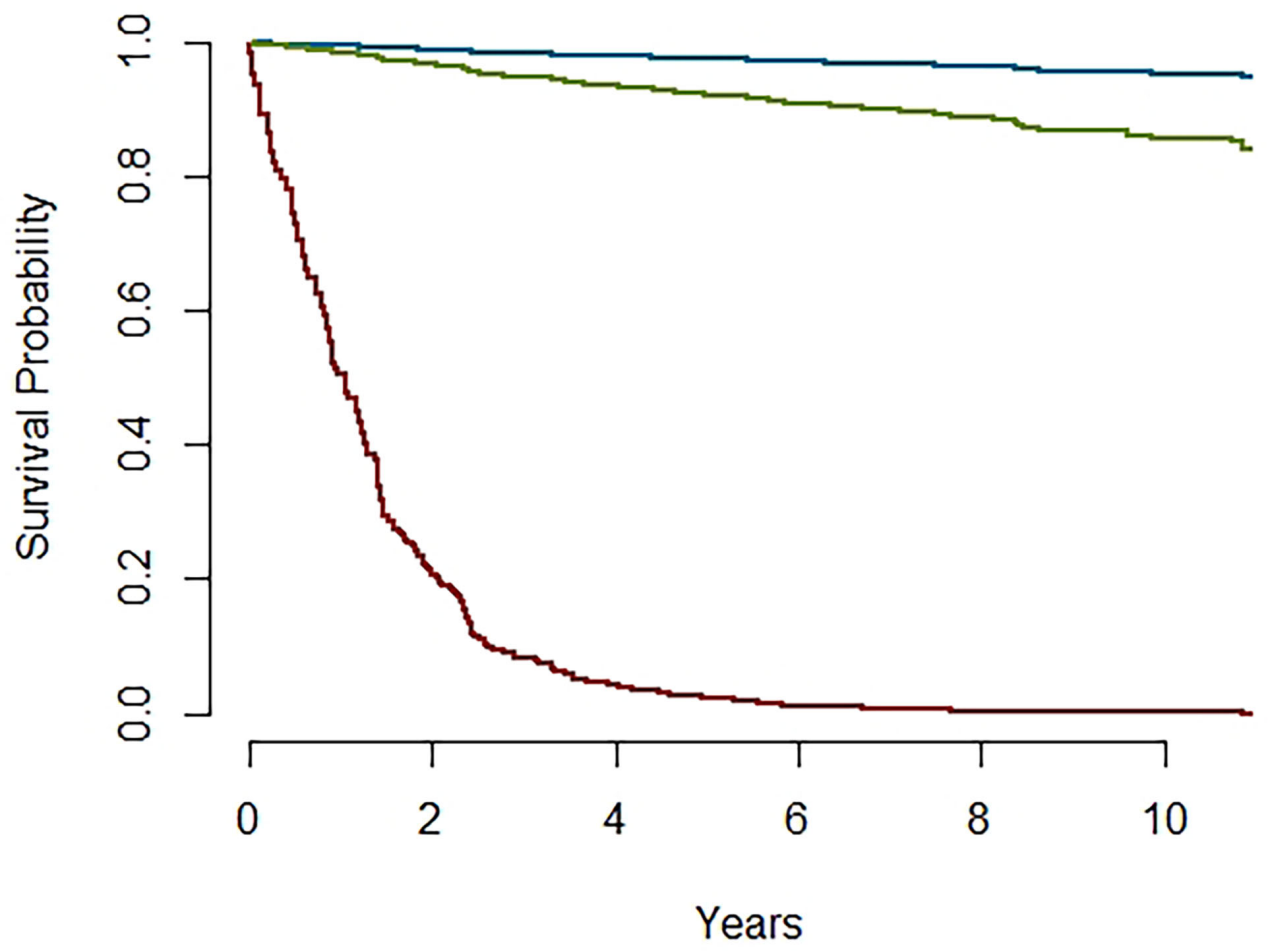
Survival probabilities for Higher (red) and Lower (blue) risk profiles and reference category (green).

The predictions of survival probabilities according to the model are presented in **Figure 6** for a high-risk profile (red line) and low-risk profile (blue line), compared to the reference category (green line). They reveal significant differences between high-risk and low-risk patients, while reference patients have a similar behavior to low-risk patients for the first 2 years since diagnosis.

## Discussion

Changes in patient management and survival in patients with early-stage NSCLC may have brought about the majority of the lung cancer long survivors. Several studies have demonstrated that curative-intent surgery, when coupled with regional lymph node examination, is generally associated with the best long-term OS in patients with early-stage NSCLC (11).

The median survival in our cohort was 63.7 months (95% CI, 56.7-64.4). Significant differences were observed in survival in our patients. In our cohort, female gender is associated with greater survival, as previously reported in other studies (12, 13). In relation to smoking habit, approximately 88% of our patients diagnosed with stage I and II NSCLC were current or former smokers and survival was significantly lower in former or current smokers at diagnosis, compared to non-smokers. These findings support the idea that tobacco is the main cause of this type of tumour (14, 15). Of note, older age is usually associated with lower current smoking and higher former smoking prevalence (16), which could explain the lower odds for adverse outcome in current compared with former smokers. In any case, these results suggest that all levels of smoking exposure are likely to be associated with lasting and progressive lung damage (17), and therefore, anti-tobacco measures should be reinforced to reduce tobacco consumption, especially among young people (18, 19).

Very significant differences were also observed in survival among treatments. For stage I–II NSCLC patients medically fit for surgery, surgical resection remains the treatment of choice, yielding the best potential choice of cure for these patients. Lobectomy was performed in 88% of our patients, matching with the current standard procedure (20). Median age at diagnosis (60.6 years) and ECOG-PS 0 or 1 among the majority of our patients are consistent with general population candidates for surgery. In addition, hospital volume affects five-year survival. In an analysis of over 2000 patients from the Surveillance, Epidemiology, and End Results (SEER) Program database, five-year survival was better among individuals undergoing resection at high-volume institutions (44 versus 33 percent at low-volume centers) (21). Our institution, being a tertiary hospital with a high volume of thoracic surgery procedures, may explain the significantly lower perioperative mortality rates, compared to those performed at lower-volume institutions (only 8% of our patients died within the first year since diagnosis).

After years of research evaluating the benefit of adding systemic therapy to surgery, two-phase III trials (8, 9) have shown an absolute survival benefit of 12 to 15% with the use of adjuvant chemotherapy in patients with stage I and II NSCLC (22). Results from The Lung Adjuvant Cisplatin Evaluation (LACE) meta-analysis demonstrated a 5.4% absolute survival benefit at 5 years [HR: 0.89 (95% CI: 0.82–0.96, p=0.005)] (4). Although in general the adjuvant studies in NSCLC have discordant results, recent data from recent studies demonstrate the clear benefit of adjuvant chemotherapy after surgery with an absolute increase in survival of 4% at five years (23). Adjuvant chemotherapy with a platinum doublet has become standard treatment for resected lung cancer patients. Of the 505 patients included in our study, 20.4% received adjuvant treatment after surgery which was slightly lower than in other similar cohorts (24–26), maybe due to the high proportion of patients with stage I versus stage II (62.8% vs 37.2%, respectively). Patients who received chemotherapy after surgery had a median survival of 65 months (IQR= 48.8) compared to 62.4 months (IQR= 52.5) for the patients who underwent surgery alone in our cohort.

Disease relapse also stands as a pivotal survival factor. Risk of local recurrence increases with the stage in lung cancer, but even stage I patients experience local recurrence up to 19% of the time (27). There was statistical difference (p=0.0085) in DFS between stage I and II in our cohort, being the median DFS for stage I 98.87 months (8.23 years) and 81.07 months (6.76 years) for stage II. The 5-year DFS for the stage I cohort was 63% and for stage II group was 48%. Of note, 32.3% (163) of our patients relapsed, 20% stage l and 28% stage ll. Of those, 40 (24.5%) had received surgery plus adjuvant treatment and 123 (75.5%) surgery alone. The possibility of identifying patients with high-risk for recurrence following surgical resection can help with surveillance plans and potentially personalize adjuvant therapy for these patients (28–30).

Prediction models are usually developed to guide healthcare professionals in their decision-making about further treatment management and to inform patients about their risks of having (diagnosis) or developing (prognosis) a particular disease or outcome. The tumour, node, and metastasis (TNM) classification is currently considered gold standard for NSCLC prognostication despite standing as a poor predictor of overall survival, accounting for less than half of prognostic variance (31). NSCLC patients are inherently heterogeneous, and their prognosis relies on many different factors, which is why accurate survival beyond TNM stage should be obtained with the development of prediction models that can obtain specific patient profiles accounting for a range of predictive factors. While different models have been published in the last years, none have demonstrated superior performance, applicability or global utility yet (31–33).

The goal of our study was to develop a clinically useful prognostic model based on currently available risk factors. Our model identified two patient risk profiles based on six discriminative factors (sex, age at diagnosis, smoking habits, stage, treatment, and performance status): a high-risk that allows identifying those patients who may benefit from adjuvant treatment or immunotherapy if they are not fit for chemotherapy; and a low-risk, that endorse adjuvant treatment in post-surgical patients who are fit for chemotherapy. It also allows adapting surveillance plans for each risk profile and avoids unnecessary tests or visits.

Our study had some limitations. First, the sample size of our cohort may have limited the significance of the results. Our model may benefit from being developed and validated with a larger cohort of patients. Furthermore, information on genomic characteristics of the patients was not provided, which may improve this model, even in early stages.

With additional prospective and multisite validation, this prognostic model could potentially serve as a predictive decision support tool for deciding the use of adjuvant treatment in early stage lung cancer.

## Conclusions

The results of the present study found that, overall, adjuvant chemotherapy was associated with the best long-term OS in patients with resected NSCLC. Age, stage and ECOG-PS were also significant factors to take into account when making decisions regarding adjuvant therapy. Continued work on individualized risk stratification, including prospective studies and research that incorporates this kind of prognostic models such as the one presented in this study as measures of risk, and is needed to better inform oncologists’ decision-making regarding adjuvant therapy use after resection achieving a personalized care in practice standard.

## Data availability statement

The original contributions presented in the study are included in the article/**Supplementary Material**, further inquiries can be directed to the corresponding author/s.

## Ethics statement

The study was approved by the Ethics Committee at Hospital Universitario Puerta de Hierro-Majadahonda (No. PI 148/15) and was carried out in accordance with the Helsinki Declaration. Written informed consent was obtained from all patients’ prior enrolment in the study.

## Author contributions

MT, MP, and PS participated in the Conceptualization, Methodology, Investigation, Writing- and Original draft preparation; GG performed the formal analysis, Data curation, Validation, Writing- Reviewing and Editing; FF participated in the Conceptualization and Investigation; RH contributed to the Investigation, Writing- Reviewing and Editing; CP performed the Data curation and Validation; AS participated in the Data curation process, Validation, and Software; JC-C contributed to the Investigation, Writing- Reviewing and Editing; JP made critical revisions on the manuscripts and provided expert opinions on implications on the study findings. All authors contributed to the article and approved the submitted version.
