# Supplementary material for: Clinical factors influencing long-term survival in a real-life cohort of early stage non-small-cell lung cancer patients in Spain
Source: Front Oncol. 2023 Feb 23;13:1074337. doi: 10.3389/fonc.2023.1074337 (PMC9996278; doi:10.3389/fonc.2023.1074337)
Supplement: Supplementary file 1 [file DataSheet_1.pdf]

SUPPLEMENTARY MATERIAL

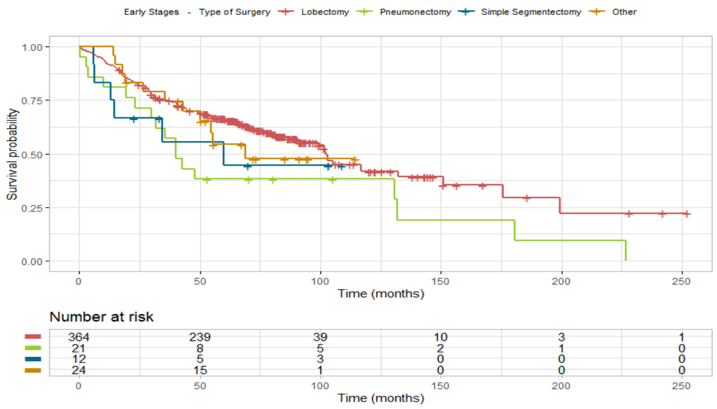

Figure S1. Survival differences according to type of surgery procedure.

Adjuvant treatment	
**Chemotherapy scheme**	
Cisplatin-Pemetrexed	2
Unknown	5
Carboplatin-Docetaxel	1
Carboplatin-Paclitaxel	29
Carboplatin-Pemetrexed	3
Carboplatin-Vinorelbina	15
Cisplatin-Docetaxel	1
Cisplatin-Etoposide VP16	1
Cisplatin-Gemcitabina	1
Cisplatin-Vinorelbine	44
Gefitinib	1
Vadimezan (ASA404)-Cisplatin-Vinorelbine	1
**Total**	**104**
**Type of toxicity during chemotherapy**	
Neutropenia	6
Febrile neutropenia	5
Anemia thrombocytopenia	4
Neurotoxicity	3
Nephrotoxicity	1
Hepatotoxicity	1
Emesis	7
Asthenia	5
Other toxicity	15
**Total**	**30**

Table S1. Type of adjuvant chemotherapy schemes and toxicities developed.
